# 3D visualization of uterus and ovary: tissue clearing techniques and biomedical applications

**DOI:** 10.3389/fbioe.2025.1610539

**Published:** 2025-07-07

**Authors:** Qiqi Liu, Zhuo Song, Simin Liu, Zirui Dong, Xi Zheng, Tak Yeung Leung, Xiaoyan Chen

**Affiliations:** ^1^ Department of Obstetrics and Gynecology, Faculty of Medicine, The Chinese University of Hong Kong, Shatin, China; ^2^ Maternal-Fetal Medicine Institute, Department of Obstetrics and Gynecology, Shenzhen Baoan Women’s and Children’s Hospital, Shenzhen University, Shenzhen, China; ^3^ The First School of Clinical Medicine, Southern Medical University, Guangzhou, China

**Keywords:** tissue clearing, 3D visualization, ovary, uterus, spatial omics, AI

## Abstract

Recent advancements in tissue clearing and three-dimensional (3D) visualization technologies have enabled subcellular-level examination of entire organs, particularly in complex structures such as the ovary and uterus. Traditional histological approaches are limited by two-dimensional views, which restrict our understanding of female reproductive system functions. In this review, we highlight the innovations in 3D tissue clearing techniques applied to uterine and ovarian tissues, which, combined with analytical tools, facilitate comprehensive 3D visualization and image analysis. We evaluate the advantages and disadvantages of three primary categories of tissue clearing techniques: organic solvent-based, hydrogel-based, and hydrogel-embedded methods, specifically regarding the uterus and ovary. Light-sheet and multiphoton microscopy complement these techniques, providing unprecedented capabilities for high-resolution imaging of large tissue volumes. Tissue clearing technologies provide a robust strategy for early diagnosis of uterine and ovarian pathologies. Additionally, we explore the integration of tissue clearing technologies with spatial transcriptomics and AI-driven analytical tools to achieve comprehensive 3D molecular mapping. We hope this review contributes to a better understanding of tissue clearing techniques and can help researchers in navigating methodological choices for uterine and ovarian investigations.

## 1 Introduction

The ovaries and uterus serve as essential components of the female reproductive system. Ovarian functions include generating mature oocytes and secreting hormones such as estrogen and progesterone (Fan et al., 2019). The uterus facilitates mammalian embryo and fetal implantation, development, and growth (Mori et al., 2023). Traditional histological approaches, limited to two-dimensional sections, fail to capture the dynamic 3D spatial relationships essential for understanding complex biological processes. Comprehensive investigation of biological progress and mechanisms requires examining intact tissues in 3D spatial views rather than sections.

Recent advances in tissue clearing, including iDISCO, ScaleA2, CUBIC, and CLARITY, address the challenges of tissue opacity and light scattering (Renier et al., 2014; Malki et al., 2015; Pinheiro et al., 2021; Tomer et al., 2014). Furthermore, significant progress has occurred in imaging technologies, including light-sheet fluorescence microscopy (LSFM) and multiphoton microscopy (MPFM). These techniques enable comprehensive and high-resolution visualization of intact large samples at cellular resolution in 3D levels (Power and Huisken, 2017; Mohler et al., 2003).

Tissue clearing techniques provide valuable insights into ovarian and uterine structure and molecular mechanisms. When combined with spatial transcriptomics, these methods enable detailed visualization of ovarian follicular architecture while simultaneously mapping gene expression gradients, revealing the complex relationship between follicular development and surrounding stromal cells. Similarly, 3D imaging integrated with single-cell analysis offers comprehensive characterization of the myometrium and its molecular regulation during pregnancy and childbirth (Kagami et al., 2020). These visualization strategies have enhanced our understanding of the cellular microenvironment, providing insights into the female reproductive system. Furthermore, these approaches are crucial in examining communication between the developing embryo and maternal tissues, offering precise comprehension of processes associated with implantation and placenta formation (Kagami et al., 2017).

This review summarizes ovarian and uterine development and emphasizes the transition from traditional techniques to 3D imaging and data analysis with commercial visualization software. We also explore future directions for integration with single-cell and spatial omics technologies. Special attention is given to AI-assisted techniques for analyzing 3D imaging data, highlighting their significance in improving the accuracy and efficiency of these studies.

## 2 Uterus and ovary

### 2.1 Development of the uterus and ovary

The female reproductive system comprises external and internal genitalia. The external genitalia include the labia majora and minora, clitoris, and vestibule, whereas the internal genitalia include the ovaries, fallopian tubes, uterus, cervix, and vagina (Septadina, 2023). In vertebrates, primordial germ cells (PGCs) are gamete precursors. They originate from the epiblast and, upon migration to extraembryonic regions, undergo fate determination, representing a critical event in embryonic reproductive system establishment.

PGC specification commences during the third week of embryonic development, and by the fourth week, these cells are identifiable in the yolk sac near the allantois (Guglielmo and Albertini, 2013). They subsequently migrate into the endodermal epithelium of the hindgut, detach from the gut wall, and reach the gonadal ridge through the dorsal mesentery (Soto-Suazo et al., 2005). In XX embryos lacking SRY gene expression, factors such as WNT4, RSPO1, and FOXL2 promote the differentiation of the bipotential gonad into an ovary, with the gradual formation of cortical and medullary compartments. PGCs that settle in the cortical region initiate meiosis and arrest at prophase I, forming primary oocytes. These are surrounded by pre-granulosa cells to establish primordial follicles (Baetens et al., 2019).

Folliculogenesis advances from the primordial stage through the primary and secondary stages, the latter characterized by multiple granulosa cell layers and steroidogenic theca cells. After puberty, periodic surges of follicle-stimulating hormone (FSH) and luteinizing hormone (LH) drive the development of a subset of follicles into the antral stage, marked by the formation of a fluid-filled cavity (Palumbo et al., 1994; Stringer et al., 2023).

The female reproductive tract primarily originates from the paramesonephric (Müllerian) ducts, which develop through cranio-caudal invagination of the coelomic epithelium adjacent to the Wolffian ducts (Orvis and Behringer, 2007). The fallopian tubes form from the upper and middle segments of the majority of the female reproductive tract, which derives from the paramesonephric (Müllerian) ducts with their ventral openings developing from the invaginated ends. During approximately the tenth week of gestation, the lower portions of both ducts merge to form the uterus and the upper portion of the vagina (Habiba et al., 2021). Considering the intricacies of the female reproductive system, we will focus on the application of tissue clearing techniques to the uterus and ovaries, aiming to reveal their underlying molecular and structural mechanisms.

### 2.2 Histologic methods for the analysis of the ovary and uterus

A comprehensive and high-resolution understanding of ovarian and uterine development is fundamental. The microscope’s invention transformed anatomical studies to the cellular level, necessitating organ sectioning for tissue examination. The primary method for visualizing the uterus and ovaries involves tissue sectioning and histology (Sarma et al., 2020; Pascolo et al., 2019; Pichat et al., 2018). For nearly a century, whole-organism analysis has relied on serial sectioning combined with histological staining to obtain a 2D perspective of 3D imaging. Histology remains widely utilized across biological disciplines, and modern techniques enable the reconstruction of 3D images from histological sections (Kartasalo et al., 2018; Berg et al., 2019).

Follicle count quantification methods employ age-special correction factors for representative numbers. This approach requires considerable effort, with results varying based on slice thickness precision, assessed slice statistics, and the research-specific correction factors (Berg et al., 2019). Advanced resolution methods, such as those developed by Lutton et al. (Lutton et al., 2017), enable myometrium reconstruction with approximately 50 µm per voxel side resolution. This facilitates specific imaging of smooth muscular tissue formation and fibrous microarchitecture within the uterus. These reconstructions correspond directly with original histological slides, enabling detailed examination of functional anatomical context in 3D images (Lutton et al., 2017). While stereology offers greater precision, its widespread adoption is limited by specialized equipment requirements and expertise needs. Furthermore, these methods are constrained by tissue distortion during sectioning and sectioning techniques inconsistencies (Gurcan et al., 2014).

### 2.3 Challenges in visualizing the intact uterus and ovary

Ovaries contain multiple tissue types, including epithelial and connective tissues, vasculature, and nerve fibers. These encompass various components, including membranes, nuclei, lipids, proteins, collagens, blood, and tissue fluids. The uterus comprises three distinct histological layers: mesometrium, myometrium, and endometrium. The mesometrium, the outermost layer, interfaces with the visceral peritoneal layer (Bates and Bowling, 2013; Taylor and Gomel, 2008). The intermediate myometrium layer contains three smooth muscle layers. Intact organ imaging presents multiple challenges: requiring long-working-distance microscopes and objectives for large samples, substantial computational resources for extensive datasets, and preservation of antigenicity, fluorophore integrity, and tissue morphology in cleared specimens (Dodt et al., 2007). Tissue clearing techniques, combined with multiphoton microscopy, light-sheet microscopy, and spinning disk confocal microscopy (SDCM), address these challenges, enabling comprehensive 3D examination of entire organs and organisms without mechanical sectioning. The non-uniform distribution of light-absorbing and scattering molecules within tissues can cause uneven light scattering, resulting in tissue opacity (Tuchin, 2015). Optical clearing techniques enhance transparency by equalizing sample RI through component modification, removal, or substitution. This adjustment reduces light scattering, improving optical transparency (Tainaka et al., 2016; Ryu et al., 2022). The application of 3D imaging for complete embryos, organs, and adult bodies has expanded through improvements in computational analysis, optical techniques, and image rendering. (Vieites-Prado and Renier, 2021; Zhu et al., 2013; Susaki et al., 2015).

## 3 Tissue-clearing methods optimized for uterus and ovary imaging

**FIGURE 1:**
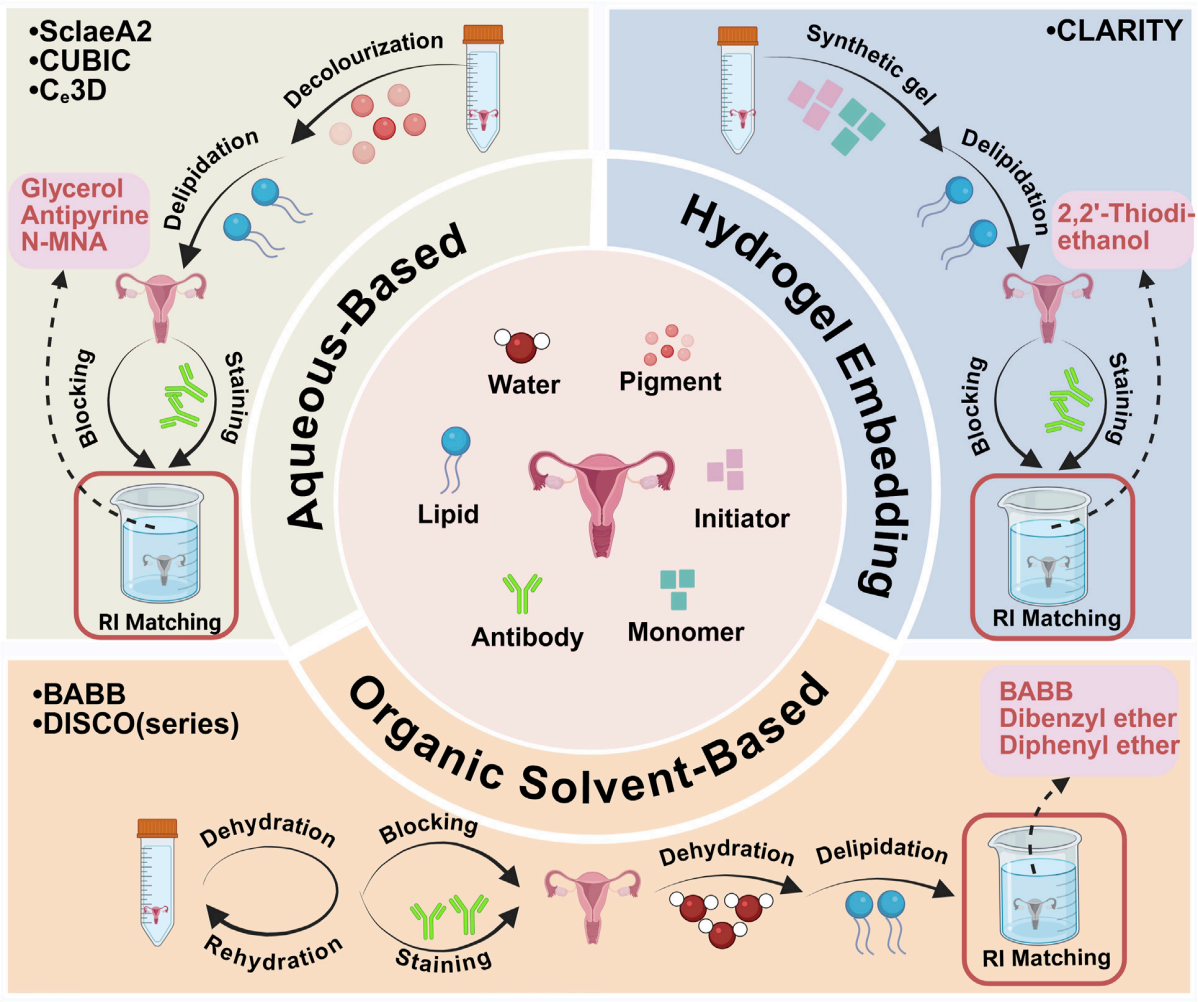
Major tissue clearing methods are applied to ovarian and uterus tissues. Created with BioRender.com.

The achievement of tissue transparency in ovarian and uterine samples is crucial for reproductive biology and pathology research, enabling detailed structural and cellular dynamic analysis. Recent technological advances have significantly improved tissue transparency methods and instruments. Here, we summarize the tissue clearing methods applied to ovarian and uterine tissues (Figure 1; Table 1) and recent developments in tissue-clearing reagents (Table 2).

**TABLE 1:** Tissue clearing methods used for ovary and uterus tissues.

	Tissue method	Sample	Microscope used	Imaging structure	Data analysis	Reference
Organic solvent based	BABB	Mouse ovaries	LSCM	Cell death in ovarian follicles	—	Zucker and Jeffay (2006)
	BABB	Mouse ovaries	LSCM	Spatial analysis of ovarian follicles	MATLAB	Faire et al. (2015)
	BABB	Mouse ovaries	OPT	Lymphatic vessels in ovaries during late gestation	—	Svingen et al. (2012)
	BABB	Mouse uterus	LSCM	Uterine gland reoriented with implantation	Imaris	Arora et al. (2016)
	BABB	Mouse uterus	LSCM	Uterine folding during early pregnancy	Imaris	Madhavan et al. (2022)
	3DISCO	Mouse uterus	LSFM, MPFM	Topography of crypts and glands during implantation	Imaris	Yuan et al. (2018)
	iDISCO	The ovaries of zebrafish, rainbow trout, and mouse	LSCM	Oocyte counts	FIJI, Cellpose	Lesage et al. (2023)
Aqueous based	ScaleA2	Mouse ovaries	LSCM	Oocyte and germ cell counts	Imaris	Malki et al. (2015)
	ScaleA2	Mouse uterus	LSFM	Mouse uterine gland morphology	Imaris	Vue et al. (2018)
	ScaleA2	Mouse uterus	LSFM	Mouse uterine epithelial morphogenesis	Imaris	Vue and Behringer (2020)
	CUBIC	Mouse ovaries	LSFM, LSCM	Individual oocytes in the developing follicles	ZEN	Kagami et al. (2018)
	CUBIC	Mouse ovaries	LSFM	Ovarian vasculature and innervation	Imaris	Tong et al. (2020)
	CUBIC	Mouse ovaries	LSCM	Oocyte counts and meiotic initiation	Imaris	Soygur et al. (2021)
	CUBIC	Mouse uterus	LSFM	Quantification of endometrium and myometrium	Imaris	Liu et al. (2023)
Hydrogel embedding	CLARITY	Mouse ovaries	LSCM	Follicle counts and vasculature	Fiji, Imaris	Feng et al. (2017)
	CLARITY	Mouse ovaries	LSFM	Follicles counts	Imaris	Ma et al. (2018)
	Ce3D	Mouse ovaries	LSCM	Ovarian angiogenesis	Fiji, Imaris	Xu et al. (2022)
Combined	BABB, iDISCO	Mouse ovaries	LSCM	Spatial analysis of ovaries	Imaris, MATLAB	Soygur et al. (2023)
	CUBIC, ScaleA2	Mouse ovaries	MPFM	Oocyte/follicle quantificition	Imaris	Boateng et al. (2021)
	iDISCO, CUBIC	Mouse ovaries	LSFM, SDCM	Growing follicles, oocytes, vasculature	Imaris	McKey et al. (2020)
	CLARITY, ScaleA2	Mouse ovaries	LSFM	Blood and lymphatic vessels	Imaris	Oren et al. (2018)

LSCM, laser scanning confocal microscopy; LSFM, light-sheet fluorescence microscopy; MPFM, multi-photon fluorescence microscopy; OPT, optical projection tomography; SDCM, scanning disk confocal microscopy.

**TABLE 2:** Tissue-clearing reagents and signal preservation for ovary and uterus imaging.

Clearing method	Dehydration	Delipidation	Staining	FP signal	Decolorizing	RI matching
BABB	Ethanol	Benzyl alcohol	Quickly diminished fluorescence signals	Major loss	—	BABB
3DISCO	Tetrahydrofuran	Dichloromethane	Diffusion-limited staining	Major loss	—	Dibenzyl ether
iDISCO	Methanol/Tetrahydrofuran	Dichloromethane	Dehydration-rehydration process by MeOH and PBS	Major loss	H_2_O_2_	Dibenzyl ether
uDISCO	Dichloromethane	Dichloromethane	Preserving endogenous fluorophore	Preserved	—	Diphenyl ether
ScaleA2	—	Triton X-100	Urea induces molecular flux	Preserved	Quadrol	Glycerol
CUBIC	—	1,2-Hexanediol/Triton X-100	Diffusion staining	Modest loss	1-Methylimidazole/Quadrol	Antipyrine/N-Methylnicotinamide (MNA)
C_e_3D	—	Triton X-100	Permeabilization flow cytometry buffers	Preserved	N-Methylacetamide	N-Methylacetamide and Histodenz
CLARITY	—	Sodium dodecyl sulfate	Diffusion-limited staining	Preserved	N,N,N′,N′-Tetrakis	2,2′-Thiodiethanol

### 3.1 Organic solvent-based tissue clearing

Organic solvent-based tissue clearing techniques provide superior tissue transparency by harmonizing components RIs via high-RI organic solvents. These protocols primarily involve tissue dehydration, lipid removal, and sample infusion with clearing solution to standardize remaining structures refractive indices. Solvent-based tissue clearing techniques compatible with immunofluorescence staining, including benzyl alcohol/benzyl benzoate (BABB) and 3D imaging of solvent-cleared organs (DISCO), represent preferred approaches for ovarian and uterine tissue clearing (Combes et al., 2009; Svingen et al., 2012; Faire et al., 2015; Dodt et al., 2007). These techniques offer cost-effectiveness and enable sample clearing within several days.

To investigate the dynamic morphogenesis of entire organs, Zucker and colleagues employed BABB alongside advanced microscopy techniques to examine whole mouse ovaries, focusing on morphology, apoptosis detection, and spectroscopy (Zucker and Jeffay, 2006). The application of LysoTracker Red to fresh, intact mouse ovaries enables identification of apoptotic cells. Tissue morphology was preserved through fixation with 4% paraformaldehyde and 1% glutaraldehyde, enhancing the background fluorescence signal and facilitating overall morphological visualization. This methodology emerged from the unexpected observation that oocyte nucleus volume increases proportionally with follicle growth, enabling differentiation between primordial and growing follicles, resulting in unprecedented quantification accuracy.

Beyond its quantification and spatial analysis capabilities, whole-mount imaging excels at detecting rare events. Whole-mount immunofluorescence staining, enhanced through BABB clearing, provides 3D visualization of the uterus and its adaptive responses to embryo implantation (Arora et al., 2016). The researchers refined the BABB protocol through meticulous uterine washing followed by incubation with fluorescently labeled secondary antibodies, specifically Alexa Fluor IgGs, to enhance protein and structural visibility within the tissue. Although BABB was initially utilized as an organic solvent for clearing mouse organs, it proved insufficient for larger samples due to rapid fluorescence signal degradation during alcohol-based dehydration. These limitations were addressed by replacing BABB with a combination of dibenzyl ether (DBE) and tetrahydrofuran (THF), known as 3DISCO solvent-based clearing, and iDISCO, which incorporates immunolabeling (Renier et al., 2014; Ertürk et al., 2012). The iDISCO method, developed by Renier et al. (Renier et al., 2014), enables whole-mount immunolabeling and volumetric imaging of mouse embryos and adult organs. In contrast to 3DISCO, uDISCO facilitates comprehensive whole-body clearing and imaging while preserving endogenous fluorescent proteins for extended periods (Pan et al., 2016; Li et al., 2018).

Within these adaptations, iDISCO application to adult ovaries achieved effective fluorescence signaling and deep antibody penetration, facilitating detailed visualization of follicular structures and the ovarian interstitial compartments in adult mice (McKey et al., 2020). McKey and colleagues examined morphogenetic events in mouse ovary development, discovering that ovary encapsulation correlates with mesonephric duct expansion and regional differentiation into the oviduct, utilizing tissue clearing and light sheet microscopy (McKey et al., 2022). Yuan and colleagues documented 3D visualization techniques revealing distinct embryo‒gland interactions within the crypt, implementing a modified 3DISCO method for tissue clearing. Through whole-mount immunostaining, 3DISCO clearing, and light-sheet imaging, they highlighted HB-EGF ’s essential role in blastocyst-gland communication (Yuan et al., 2018). Despite solvent-based tissue clearing methods demonstrating excellent clearing performance and enabling whole-body imaging at subcellular resolution, certain limitations persist, including significant sample shrinkage, organic solvent toxicity, and fluorescent protein quenching (Tian et al., 2021).

### 3.2 Aqueous-based tissue clearing

Aqueous-based tissue clearing methods are classified into two main categories: (a) simple immersion and (b) hyperhydration. Simple immersion involves submerging biological samples in an aqueous solution to match refractive indices, enabling gradual clearing. Common agents include sucrose (Tsai et al., 2009), fructose (Costantini et al., 2015; Ke et al., 2013), glycerol (Meglinski et al., 2004), 2,2′-thiodiethanol (TDE) (Aoyagi et al., 2015; Hou et al., 2015; Staudt et al., 2007), and formamide (Kuwajima et al., 2013). While simple immersion in aqueous clearing solutions preserves lipid structures, it inadequately clears large tissues containing connective tissues such as ovaries and uterus. An alternative method involves delipidation and reducing organ RI throughout the clearing process. Scale pioneered this mechanism, employing detergent-based lipid removal with urea-mediated hydration (supported by glycerol) for tissue clearing (Hama et al., 2011). This approach removes lipids using non-hydrophobic solvents to maintain an aqueous environment suitable for fluorescent proteins, requiring extended incubation periods of days to months with detergents such as Triton X-100, necessitating frequent solution changes (Hua et al., 2008).

When combined with sucrose preincubation, ScaleA2 enables oocyte imaging via TRA98 in fetal ovaries, particularly during meiosis and oocyte attrition (Malki et al., 2015). Between E15.5 and E18.5, total germ cell numbers decreased by approximately 27%, a smaller decline than the nearly twofold reduction observed in histological samples (Malki et al., 2014). ScaleA2 and TRA98 immunofluorescence were also employed to examine asymmetries between right and left ovaries regarding ovulation rates, confirming previous findings (Wiebold and Becker, 1987). Similar to the findings of Faire et al. (Faire et al., 2015) regarding adult ovaries, detailed analysis revealed no significant differences in germ populations between right and left ovaries at E15.5 and E18.5. This research established a framework for 3D analysis of embryonic ovaries, offering a comprehensive analysis pipeline, documenting error rates, and comparing sampling and whole-mount approaches directly (Malki et al., 2015).

The ScaleA2 technique was utilized to precisely visualize individual developing endometrial glands and their arrangement within the intact uterus, establishing a uterine gland morphology system during development comprising five stages: Bud, Teardrop, Elongated, Sinuous, and Primary Branch (Goad et al., 2017; Vue et al., 2018). Vue et al. (2018) tracked the morphological transformations of uterine glands from postnatal day 0 to day 21, revealing clear stages of gland development. Initially, at birth (P0), no glands were evident; however, by P8, exposed buds and teardrop-shaped epithelial invaginations were monitored (Filant and Spencer, 2014). This staging system uses a standardized method for reviewing and measuring prepubertal uterine gland morphology, enabling more comprehensive investigations into uterine gland growth and pathology (Vue et al., 2018). The researchers enhanced ScaleA2 for 3D imaging of perinatal mouse uterine glands, including structures such as the uterine orbit and ventral ridge. Their analysis revealed the specific temporal patterns of uterine gland formation, demonstrating that uterine adenogenesis initiates at P4, with postpartum uterine epithelial folds appearing by P5. These observations indicate that uterine morphological development exhibits greater complexity during the perinatal period than previously recognized (Vue and Behringer, 2020).

The clear, unobstructed brain/body imaging cocktails and computational analysis (CUBIC) protocol represents an innovative modification of SCALE (Susaki et al., 2014; Susaki et al., 2015; Pinheiro et al., 2021). The initial reagents used in this method consist of polyhydric alcohol, Triton-X 100, and urea, which jointly help with delipidation. The developed reagent, made up of triethanolamine, polyhydric alcohol, sucrose, and urea, aligns with the RI and enhances transparency while also protecting against endogenous fluorescent signals. CUBIC has been effectively utilized to reveal the 3D structures of ovaries, employing endogenous fluorescent reporter proteins and immunolabeled structures. Kagami et al. (Kagami et al., 2018) utilized a customized CUBIC protocol to visualize common EGFP in adult mouse ovaries as evidence of concept. Incubation with the ScaleA2-CUBIC-1 reagent alone successfully cleared fetal mouse ovaries, enabling the visualization of endogenous fluorescent signals and immunolabeling (Soygur et al., 2021). McKey and colleagues employed iDISCO+ and iDISCO + CUBIC techniques to visualize intact organ dynamic morphogenesis, revealing ovary folding, encapsulation, and integration with oviduct morphogenesis during murine development from embryonic day 14.5 to postnatal day 0 (McKey et al., 2020).

To obtain stereoscopic whole images of the intrauterine murine embryo and placenta, Kagami and colleagues utilized transgenic mice (Kagami et al., 2017). Clear images of EGFP-positive embryos and placentas were recorded, confirming the precise 3D locations of invading trophoblasts at the feta-maternal interface (Kagami et al., 2017). Using this protocol, all placental tissues from pregnant mice (E14.5) were effectively cleared through the modified CUBIC protocol (Kagami et al., 2017). However, CUBIC employs extremely high levels of Triton (50%) to optimize lipid removal. This process, while effective, often leads to substantial protein loss (24%–41%), reducing epitope concentrations and possibly diminishing the effectiveness of immunostaining (Chung et al., 2013). Li et al. (Li et al., 2017) utilized clearing-enhanced 3D (Ce3D) techniques, whereby a blend of N-methylacetamide (22% wt/vol) with Triton X-100 and Histodenz (86% wt/vol) effectively renders tissues transparent. This approach not only maintains the fluorescence of reporter proteins but also supports the execution of direct multiplex antibody-based immunolabeling (Li et al., 2017). By utilizing the benefits of C_e_3D technologies, Xu and colleagues integrated tissue transparency techniques with endogenous multicolor reporter mouse models. This integration resulted in the creation of a tissue-scale 3D imaging system with single-cell resolution. This system enables detailed imaging and tracking of angiogenesis and vascular remodeling in whole ovaries and live ovarian follicles (Xu et al., 2022).

### 3.3 Hydrogel embedding tissue clearing

The aqueous-based clearing methods reviewed thus far have limitations: they can clear only small samples (as in simple immersion) or operate slowly (as with hyperhydration). Moreover, techniques that employ harsh solvents or high concentrations of detergents risk significant protein loss from the tissues. The CLARITY (clear lipid-exchanged acrylamide-hybridized rigid imaging/immunostaining/*in situ* hybridization-compatible tissue hydrogel) technique addresses these challenges by initially embedding the tissue in a hydrogel matrix (Tomer et al., 2014; Chung et al., 2013; Yang et al., 2014). In the CLARITY method, tissues are embedded in a hydrogel using acrylamide or bisacrylamide solutions, which helps stabilize proteins and maintain the structural integrity of the samples (Malkovskiy et al., 2022). Lipids not integrated into the hydrogel are removed via electrophoretic tissue clearing or passive thermal diffusion methods (Yang et al., 2023). Feng et al. (Feng et al., 2017) developed a passive CLARITY method to reach a complete level of transparency for mouse ovaries over a period of 4–8 weeks. The quantity of follicles increased approximately 300,000-fold from the primordial follicle stage to the preovulatory stage, indicating significant follicle growth and dynamic ovarian tissue remodeling (Feng et al., 2017). Spatial evaluation revealed that follicles increasingly accumulate within the ovary as folliculogenesis advances from the primordial to the antral stages, accompanied by active ovarian remodeling in each cycle, which is consistent with prior observations (Hirshfield and Desanti, 1995). Isaacson and colleagues introduced CLARITY combined with light-sheet fluorescence microscopy for rapid volumetric imaging of both male and female reproductive systems in humans (Isaacson et al., 2020). This research paves the way for future studies focused on clarifying both normal developmental processes and the genetic underpinnings of congenital urogenital anomalies.

### 3.4 Designing a tissue-clearing experiment

Significant advancements have been made in various tissue clearing methods; however, several critical factors must be considered prior to initiating this experiment, including sample integrity, size, and fluorescence attenuation.

The integrity of the sample is essential during the whole process, as this experiment involves numerous steps and lasts for several weeks. Poor sample integrity undermines the retention of proteins, antigens and macromolecules, as well as overall tissue shape and stability, leading to inferior visualization of intact tissue (Weiss et al., 2021). In hydrogel embedding methods, a polymerized hydrogel or tissue gel matrix minimizes structural damage, allowing more complete removal of lipids from the tissue while reducing protein loss and distortion (Gradinaru et al., 2018). The degree of fixation, the density of the hydrogel, and the strength of the detergent can all be adapted to strike a balance between maintaining tissue integrity and increasing optical transparency.

Larger samples, with approximate dimensions of 10 × 10 × 10 mm^3^, present even greater challenges in staining, clearing, and imaging. Additionally, tissues with heterogeneous structures demand specialized clearing strategies, such as delipidation or depigmentation (Chi et al., 2018; Pende et al., 2020). These procedures can drastically affect factors such as transgenic signal integrity, structural morphology, and nucleotide preservation. To overcome this limitation, shrinkage-prone clearing methods, such as those utilizing organic solvents, can reduce mouse bodies to approximately one-third of their original size, fitting within the microscope’s physical constraints. This is because reductions in tissue size and improvements in resolution considerably increase the data volume. Conversely, certain hydrogel embedding and aqueous-based techniques can be adapted to expand the construct isotropically for super-resolution imaging, thereby increasing the resolution (Chen et al., 2015; Ku et al., 2016).

**FIGURE 2:**
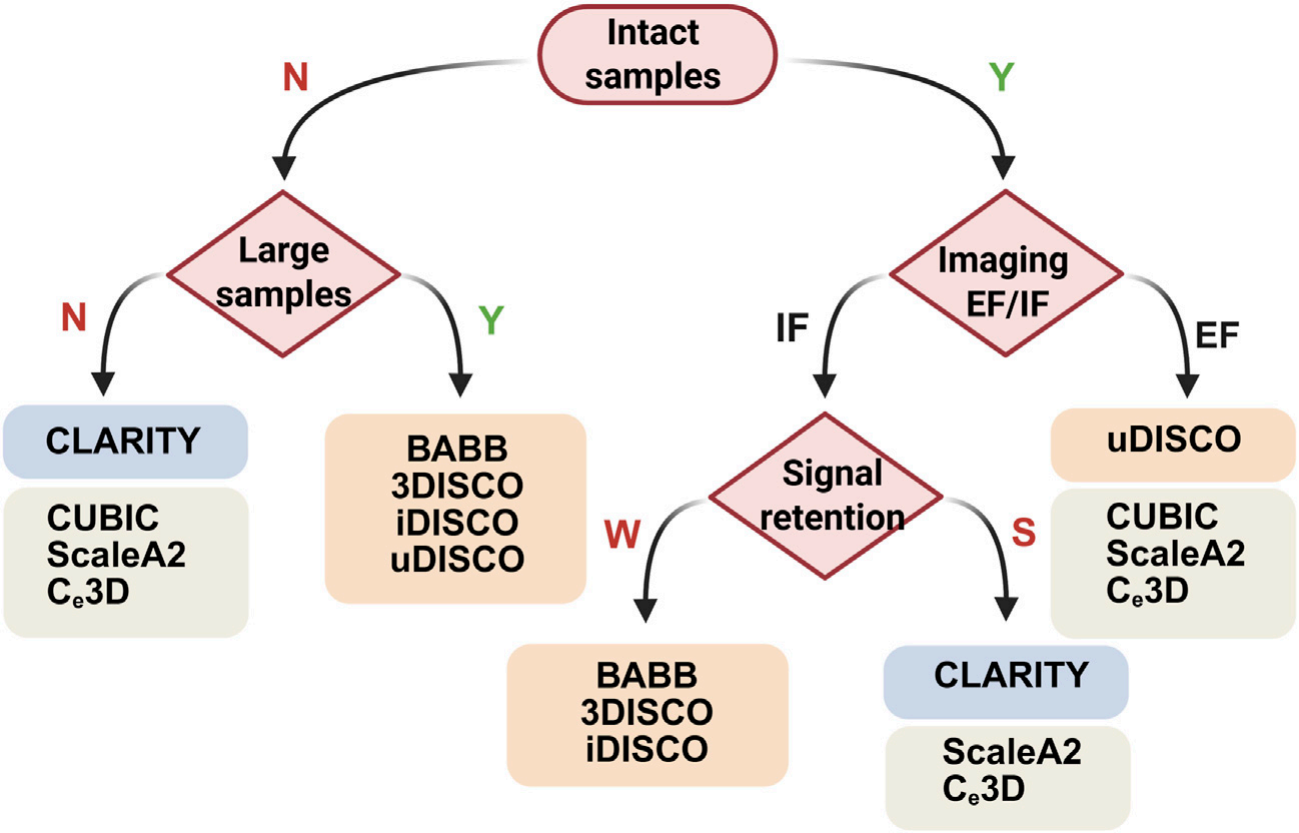
Decision trees for choosing clearing protocols on the basis of the integrity, size and fluorescence signals of samples. N, no; Y, yes; IF, immunofluorescence; EF, endogenous fluorescence; W, weak; S, strong. Created with BioRender.com.

The retention of endogenous fluorescent (EF) protein expression, together with the use of antibodies and conjugated dyes, significantly influences the choice of protocol. Adaptations to organic solvent-based clearing protocols are continuously advancing to increase fluorescence retention, primarily with changes in dehydration processes, temperature, and pH conditions (Masselink et al., 2019). Moreover, aqueous-based methods have shown success in imaging endogenous transgenic fluorescence. In most cases, a robust signal can be achieved via the use of antibodies that amplify the signal via a stable fluorescent dye. The advantages of this approach include the ability to select wavelengths that minimize autofluorescence and absorption, increase brightness, reduce bleaching, and increase compatibility with various clearing protocols. Here, the selection of an appropriate clearing protocol is based on the integrity of the sample, the sample size, and the retention of both endogenous fluorescence and immunofluorescence (Figure 2).

### 3.5 Reproducibility and cost

Recent advancements have enhanced the reproducibility of tissue clearing protocols through standardized commercial solutions and equipment-assisted methodologies. Solvent-based techniques now utilize consistently formulated chemical components, while aqueous approaches benefit from commercial systems for hydrogel polymerization and electrophoretic tissue clearing (ETC), reducing inter-laboratory variability (Jensen and Berg, 2017). Nevertheless, antibody-dependent fluorescence labeling remains a critical reproducibility challenge due to batch-to-batch inconsistencies, particularly evident in large tissues where off-target binding amplifies background noise. Mitigation requires rigorous antibody validation using uncleared sections and establishment of dedicated repositories for clearing-validated reagents. Protocol harmonization ensures experimental consistency across studies by standardizing critical parameters, including sample dimensions, solution compositions, incubation conditions, tissue deformation metrics, and antibody specifications (manufacturer, lot, concentration) (Richardson et al., 2021).

The economic viability of tissue clearing techniques is primarily governed by reagent accessibility, protocol complexity, and ancillary infrastructure demands. Solvent-based methods, such as BABB and iDISCO, utilize relatively low-cost organic chemicals (Azaripour et al., 2016). For hydrogel-embedding approaches, examples like CLARITY and SHIELD incur higher reagent expenditures due to proprietary hydrogel monomers and electrophoretic instrumentation; however, these methods offset long-term costs by minimizing tissue distortion artifacts that necessitate repeat experiments (Choi et al., 2021). Aqueous techniques, including CUBIC and SeeDB, employ moderately priced reagents compatible with standard laboratory equipment but require extended processing durations, thereby increasing operational overhead (Matsumoto et al., 2019; Ke et al., 2013).

## 4 Tissue clearing and microscopic methods for uterus and ovary imaging

Currently, with advancements in the field of fluorescence light microscopy, numerous optical sectioning techniques are available on the market, many of which excel at imaging cleared tissues. Notable methods include confocal microscopy, multiphoton fluorescence microscopy, and light-sheet fluorescence microscopy (Table 3).

**TABLE 3:** Comparison of microscopy techniques for tissue-clearing samples.

Microscopy	Principle	Advantage	Disadvantage	Reference
Laser scanning confocal microscopy (LSCM)	Point-scanning with pinhole filtering	1. High resolution and contrast 2. Three-dimensional imaging 3. Non-invasive nature	1. Limited penetration depth 2. Potential for photodamage.	Yan et al. (2022), Bohn et al. (2020), Zhang et al. (2018), Földes-Papp et al. (2003)
Multiphoton fluorescence microscopy (MPFM)	Nonlinear excitation in sub-femtoliter focus	1. Longer wavelength excitation light 2. Minimizing photodamage and photobleaching outside the focal plane	1. Lower signal-to-noise ratio 2. The depth of penetration is limited by scattering and absorption.	Cao et al. (2020), Xu et al. (2024), Horton et al. (2013)
Light-sheet fluorescence microscopy (LSFM)	Orthogonal sheet illumination and detection	1. Rapid acquisition of images 2. Excellent optical sectioning capabilities 3. Large field of view 4. Reduced phototoxicity and photobleaching	1. Optical aberrations 2. Alignment and maintenance	Jacob et al. (2024), Kafian et al. (2020), Stelzer et al. (2021)

### 4.1 Confocal microscopy

**FIGURE 3:**
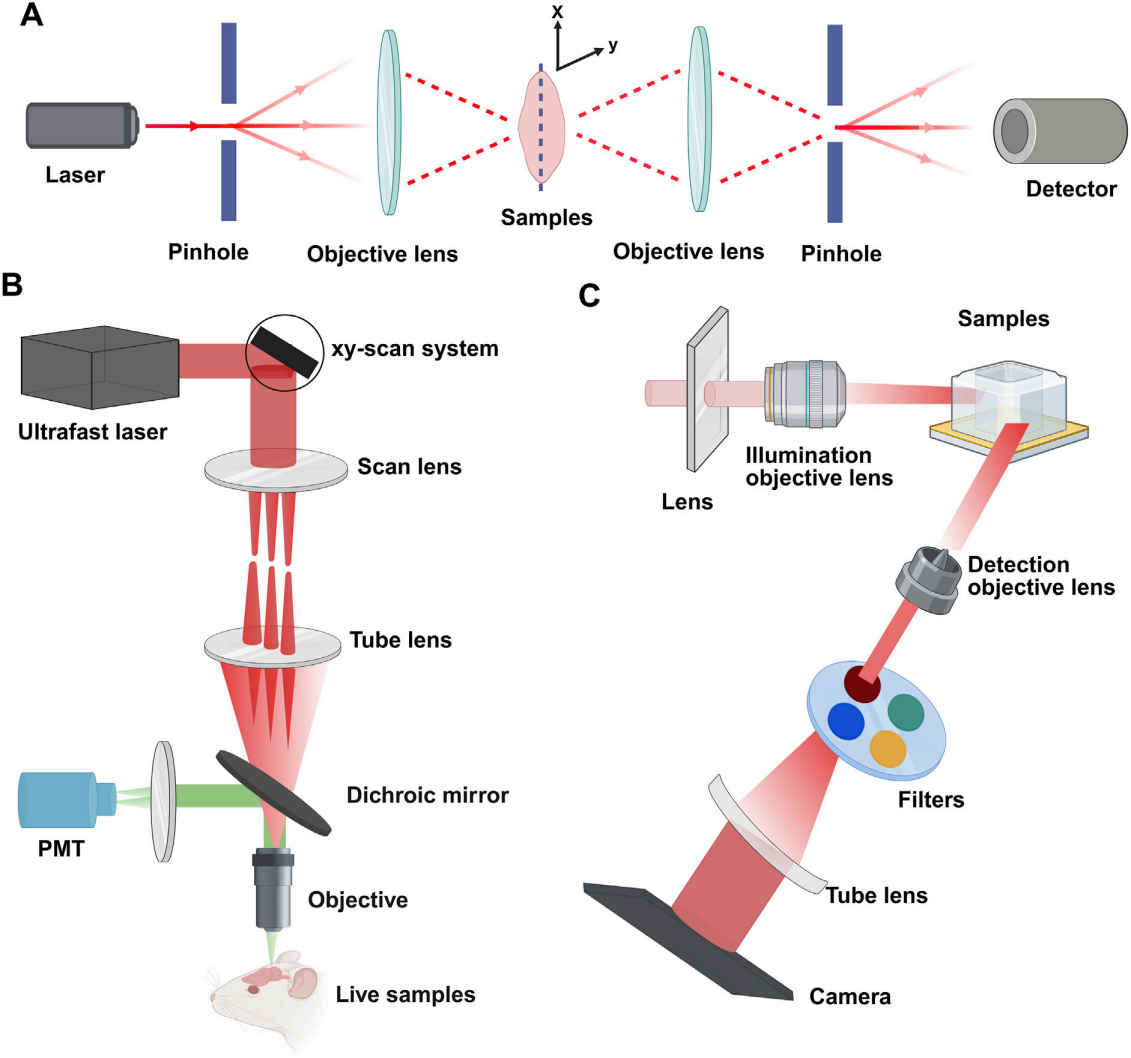
Principles of confocal, multiphoton, and light-sheet microscopy. **(A)** Confocal microscopy: a pinhole filters the illuminant to a point source focused by the objective lens. This enables optical sectioning via point-scanning in the x-y plane. Deeper scanning captures sequential images, and computer processing constructs the 3D structure. **(B)** Multiphoton microscopy: nonlinear excitation enables deeper tissue penetration. Illustrated with epi-fluorescence detection using one photomultiplier tube (PMT). **(C)** Light-sheet microscopy: a cylindrical lens forms a static light sheet illuminating the sample plane along the z-axis. Parallel illumination across the field of view minimizes photodamage. Detection occurs orthogonally (x-y plane). Created with BioRender.com.

For decades, confocal microscopy has been a staple for imaging entire developing gonads in their entirety. Laser scanning confocal microscopy (LSCM) directs a high-energy photon to illuminate a single point and employs a pinhole to filter out-of-focus light, reducing light scattering effects. Confocal microscopy eliminates scattered light artifacts by using laser point illumination focused through an exclusionary pinhole (Figure 3A). This illuminates a single focal point in the specimen, with emitted light filtered through a conjugate detection pinhole to reject out-of-focus photons. Sequential x-y-z scanning of the focal plane builds high-resolution 3D images through optical sectioning (Zhang et al., 2018; Földes-Papp et al., 2003). This technique results in high-resolution, high-contrast optical sections of the tissue (Clendenon et al., 2011). Advances in confocal imaging and 3D analysis have enabled quantitative studies of ovarian follicles and their spatial distribution within the mouse ovary; however, these techniques have yet to be fully utilized in studying the spatiotemporal dynamics of early female meiosis. Soygur et al. developed an algorithm that utilizes confocal imaging and a novel 3D quantitative approach to investigate meiotic initiation in whole intact mouse fetal ovaries (Soygur et al., 2021). This study used 3D imaging to map meiotic initiation, revealing a novel radial pattern of meiotic onset that precedes the anterior‒posterior (A‒P) wave in mouse fetal ovaries (Soygur et al., 2021). To increase the axial resolution in a confocal point-scanning system, choosing a high numerical aperture (NA) detection lens and minimizing the pinhole size are vital. Enhanced axial resolution does not just improve lateral resolution; it likewise demands capturing more images to completely cover the tissue. This causes greater light exposure and increased bleaching of the sample; for example, a 5 mm deep tissue section imaged with 5 μm z-steps in a point-scanning system would be exposed to excitation light 1,000 times (Weiss et al., 2021).

### 4.2 Multiphoton microscopy

To alleviate out-of-focus photobleaching and accomplish deeper imaging, multiphoton fluorescence microscopy (MPFM) utilizes a pulsed infrared laser to excite fluorophores via nonlinear multiphoton absorption (Denk et al., 1990). In two-photon microscopy (2PFM), simultaneous absorption of two photons confines excitation to a sub-femtoliter focal volume (<1 fL) due to quadratic intensity dependence, providing intrinsic optical sectioning without confocal pinholes (Denk et al., 1990). Three-photon microscopy (3PFM) uses longer wavelengths and cubic intensity dependence to suppress background, achieving theoretical penetration depths of 3–4 mm despite marginally lower resolution (Figure 3B) (Horton et al., 2013; Xu et al., 2024). The longer excitation wavelengths of infrared light (750–1,500 nm) decrease light scattering by at least 16-fold, dramatically enhancing the possible imaging depth (Cutrale et al., 2019). MPFM is increasingly popular for deep tissue imaging across various systems because of its low toxicity, making it the preferred method for long-term intravital imaging (Mohler et al., 2003; Scheele et al., 2022). A recent study integrated ScaleA2 and CUBIC with MPFM to investigate the maturation of the ovarian reserve and analyze the patterns of follicular attrition in the perinatal mouse ovary. While MPFM yields high-resolution images and excels over confocal microscopy in deep imaging up to approximately 1.5 mm, its compatibility with standard fluorophores is limited, and the scanning of a single excitation spot across the sample by most multiphoton instruments leads to lengthy acquisition times.

### 4.3 Light-sheet fluorescence microscopy

Light-sheet fluorescence microscopy (LSFM) employs orthogonal illumination and detection paths (conventionally x-illumination/z-detection) to project a thin laser sheet, via cylindrical lens in selective/single plane illumination microscope (SPIM) or scanned mirror in digital scanned laser microscope (DSLM) through the sample, while a perpendicular objective collects emitted fluorescence (Figure 3C) (Stelzer et al., 2021). LSFM presents a compelling option for accelerating imaging speeds and independently optimizing lateral and axial resolution (Voie et al., 1993; Huisken et al., 2004; Dodt et al., 2007; Power and Huisken, 2017). Moreover, the technique exceeds point-scanning methods in speed, significantly reduces photobleaching, and allows for adaptable sample mounting and orientation. This flexibility is beneficial in bypassing areas that are unclear in whole-mount samples. Since Dodt et al. first applied LSFM with tissue clearing in 2007, LSFM has developed into an important method for volumetric imaging of tissues (Dodt et al., 2007).

LSFM offers a broad field of view and quick image acquisition, making it possible for researchers to swiftly and successfully capture detailed signal information of whole intact samples. This ability makes it specifically fit for imaging intricate biological structures such as ovarian follicles. Lin et al. (2017) demonstrated that LSFM can effectively visualize a range of follicular features, including diameters ranging from 70 μm to 2.5 mm, sizes of developing cumulus oophorus complexes (COCs) ranging from 40 μm to 110 μm, and follicular wall thicknesses ranging from 90 μm to 120 μm. The system excels at identifying follicles at all developmental stages, particularly the small primordial follicle clusters crucial for egg nest formation. Recent advancements have enabled the use of LSFM to characterize single-cell interactions within 3D tissues, providing insights into the cellular mechanics and interactions that are crucial for understanding physiological and pathological processes in the uterus (Fredrickson et al., 2021; Tomizawa et al., 2024). For example, optical visco-elastography combined with LSFM has been used to study the maternal–fetal interface, revealing the mechanical properties and interactions between endometrial stromal fibroblasts and placental extravillous trophoblasts (Tomizawa et al., 2024).

### 4.4 Strategies for bioimaging analysis

Another critical step is to process and analyze the acquired images to extract valuable biological insights. Image adjustment primarily improves visibility for presentation, whereas analysis focuses on detailed data extraction from the dataset. Handling LSFM images involves two main phases: essential image pre-processing to address imaging process and stitch image tiles into volumetric dataset, followed by information extraction through segmentation and quantitative evaluation (Richardson et al., 2021; Delgado-Rodriguez et al., 2022).

Pre-processing resolves measurement artifacts and reconstructs accurate representations, potentially with lossless compression. This often involves deskewing to ensure voxel consistency within 3D systems. Tools range from commercial software (Imaris, AMIRA) with robust 3D analysis but high costs to open-source platforms (FiJI/ImageJ, Napari) offering free customizable operations via plug-ins (Folts et al., 2024).

Object segmentation identifies regions of interest (ROIs) based on fluorescence or size. Available through both Imaris/AMIRA and open-source tools with plug-ins like Stardist/Cellpose (Lesage et al., 2023; Stringer et al., 2021). Applications range from cellular-scale oocyte studies in mouse/medaka ovaries to organ-level vasculature/morphogenesis analyses (Belle et al., 2017; Combes et al., 2009; Bunce et al., 2021). Object classification faces ovarian challenges: size-dependent segmentation limits follicle differentiation (Folts et al., 2024).

## 5 Future directions in the female reproductive system and imaging

### 5.1 Advancing early diagnosis of uterine and ovarian pathologies

3D tissue clearing technologies overcome the fundamental limitations of traditional 2D histology and clinical imaging by enabling volumetric, subcellular-resolution visualization of intact uterine architecture. Tissue clearing technologies, such as the PEGASOS method, achieve tissue transparency through chemical treatment and integrate fluorescence labeling for subcellular-resolution 3D imaging (Jing et al., 2018). This enables spatial localization of microscopic lesions, including adenomyotic lesions, within deep tissues such as the myometrium (Martire et al., 2024). These technologies overcome the inherent limitations of 2D histopathology, which cannot reliably discern invasive growth patterns crucial for accurate tumor grading. By enabling 3D visualization of transparent tissues, such as pancreatic tumors processed using the FLASH technique, these methods reveal growth architectures and microinvasive margins that remain undetectable in traditional planar sections (Messal et al., 2019; Noë et al., 2018). This capability is particularly significant for assessing tumor margins, as 3D mapping of infiltrative borders and internal features like necrosis or abnormal vasculature provides detailed insights unattainable through 2D analysis (Almagro et al., 2021). For example, such approaches facilitate the reliable differentiation of leiomyosarcomas from benign fibroids or adenomyomas by exposing the spatial complexity of neoplastic infiltration. Unlike 2D slices, which obscure the true extent of local invasion and infiltration, 3D imaging directly captures the geometric relationship between tumor tissue and surrounding structures, offering a robust solution for precise tumor margin evaluation and enhancing differential diagnosis in clinical oncology (Guldner et al., 2016). Beyond neoplastic applications, 3D tissue clearing technologies like CUBIC have demonstrated utility in animal models, enabling whole-mount 3D imaging of murine intrauterine embryos and placentas by achieving tissue transparency (Kagami et al., 2017). This technique reveals 3D relationships between embryonic, placental, and uterine tissues—insights unattainable via conventional 2D methods—by visualizing spatial organizations of structures like embryonic membranes and placental vasculature. Such capabilities hold promise for advancing the understanding of uterine developmental anomalies, as they allow precise mapping of tissue interfaces with subcellular resolution (Arora et al., 2016). While currently validated in mice, this approach highlights the translational potential of 3D tissue clearing to improve diagnostic accuracy for human uterine malformations by exposing subtle structural deviations critical for fertility and pregnancy outcomes.

The advancement of tissue clearing methodologies and 3D ovarian cancer organoid models offers transformative potential for early and differential diagnosis of ovarian pathologies, including endometriomas, benign cysts, borderline tumors, and malignancies. In benign conditions like polycystic ovary syndrome (PCOS), a prevalent endocrine disorder impacting reproductive, metabolic, and cardiovascular health, tissue clearing enables high-resolution 3D visualization of pathological features previously inaccessible via conventional histology (Meier, 2018). For instance, Ma et al. utilized CLARITY-cleared ovaries to demonstrate that electro-acupuncture (EA) restores angiogenesis in antral follicles of PCOS-like rats, promoting folliculogenesis and ovulation (Ma et al., 2018). Subsequent CUBIC-based analysis further revealed aberrant ovarian innervation as a central pathological mechanism in PCOS, with EA exerting therapeutic effects via modulation of suprachiasmatic nucleus (SCN)-mediated neural pathways (Tong et al., 2020). In oncological diagnostics, 3D patient-derived ovarian cancer organoids replicate native tumor architecture and cellular heterogeneity, serving as physiologically relevant platforms for differential diagnosis, drug screening, and personalized therapy prediction (Clevers, 2016; Kopper et al., 2019; Maru et al., 2019). As tissue clearing methods and supporting technologies continue to progress, they are set to play an increasingly essential function in the study of ovarian diseases by enabling direct 3D validation of organoid-tumor congruence, precise mapping of cellular interactions, and identification of treatment-responsive heterogeneity (Maru et al., 2019). This synergistic integration of volumetric imaging and advanced organoid models thus provides unprecedented insights into the spatial dynamics of ovarian pathologies, accelerating biomarker discovery and refining diagnostic stratification across benign, borderline, and malignant entities.

### 5.2 Single-cell spatial omics in utero-ovarian tissues

Conventional single-cell transcriptomics and proteomics provide high-resolution molecular characterization but suffer from inherent spatial information loss due to tissue dissociation and sectioning (Zhou et al., 2023). This limitation impedes the identification of rare cell populations, such as ovarian micro-metastases or endometriosis-initiating cells, and obscures early pathological events at single-cell resolution. While laser-capture microdissection enables targeted single-cell analysis, its accuracy remains constrained when studying microscale pathological structures, including ovarian cancer micro-metastases (Ertürk, 2024).

Recent progress in spatial omics technologies, particularly sequential fluorescence *in situ* hybridization (seqFISH), combined with 3D tissue-clearing techniques has opened new avenues for resolving these challenges (Gandin et al., 2025). Tissue clearing methods like DISCO and Tris buffer–mediated retention of *in situ* hybridization chain reaction signal in cleared organs (TRISCO) have demonstrated remarkable potential to overcome these limitations by preserving both molecular integrity and 3D tissue architecture, enabling transcriptomic and proteomic analysis in intact millimeter-scale specimens (Bhatia et al., 2022; Kanatani et al., 2024). Achieving single-cell-resolution barcode detection in 3D-cleared tissues is essential for precise spatial mapping of molecular data, whereas implementing spatial omics technologies in volumetric formats is necessary to comprehensively analyze rare cellular events across entire organs. As these technologies mature, their integration with AI-driven data analysis pipelines will pave the way for dynamic 4D atlases of physiological processes like endometrial regeneration and ovarian aging (Ertürk, 2024; Cao et al., 2022). This transformative approach will provide fundamental new insights into the spatial regulation of gynecological pathologies, from their earliest cellular origins to tissue-scale manifestations, ultimately enabling more precise diagnostic and therapeutic strategies.

### 5.3 AI-based techniques for quantitative analysis

Another direction in reproductive biology will involve refining established methods for data analysis and applying AI-based approaches for quantitative analysis. The integration of AI-based analysis into image processing offers a new way to overcome bioimage analysis (Hallou et al., 2021). These AI-driven tools have overcome previous challenges by enhancing the precision of segment distinction and increasing the degree of classification contained in images. Furthermore, these methods dramatically reduce the time required for image analysis and minimize the risk of human error (Moen et al., 2019). AI-based tools are categorized into machine learning and deep learning (Moen et al., 2019; Hallou et al., 2021). Machine learning involves algorithms and statistical models that allow computers to learn from and generate predictions on the basis of data. Deep learning, a subset of machine learning, applies artificial neural networks that analyze data through a complex, layered structure, resembling the way the human brain forms connections and interprets information. All aspects of 3D bioimage analysis can be conducted manually, semi-manually, or fully automatically. Tools based on both machine learning and deep learning are instrumental in advancing our understanding of the anatomy and function of the reproductive system. The primary use of AI in image analysis of cleared reproductive system tissues involves counting ovarian follicles, a metric frequently used to assess fertility and ovarian health. Traditionally, counting follicles manually in histological sections is not only labor intensive but also prone to error. Manual counting methods often differ significantly between studies because of the absence of a universal scaling factor, leading to considerable variability in reported follicle counts (Tilly, 2003). Recently, Lesage and colleagues (Lesage et al., 2023) crafted a workflow employing open-source, cost-free, deep learning for analyzing 3D images of adult and larval medaka ovaries. They extended this approach to other typical animal models, such as zebrafish and mice. Their method segments ovarian follicles and calculates both the total number and volume of follicles. Image denoising was achieved via Noise2Void (Krull et al., 2019) within FiJI, with automated segmentation carried out via the CellPose algorithm (Stringer et al., 2021), initiated from an Anaconda command prompt. The open-source nature of these tools enhances the accessibility of this pipeline, while the adaptability of the deep learning framework allows it to be retrained for analyzing ovaries from different species.

## 6 Conclusion

Recent studies have laid the groundwork for understanding the functions of the mammalian ovary and mapping the 3D structure of the uterus. In the future, the creation of non-toxic clearing agents is expected to enhance the 3D visualization of samples, benefiting both laboratory analyses and clinical diagnostics. These advancements enable early detection of uterine and ovarian pathologies, significantly improving differential diagnosis and therapeutic stratification. The integration of spatial transcriptomics with cleared tissue imaging is poised to decode the molecular topography of reproductive organs, revealing previously inaccessible cell-cell communication networks during folliculogenesis and embryo implantation. AI-powered analytical platforms are emerging as essential tools for interpreting the multidimensional datasets generated by these integrated approaches, capable of identifying subtle pathological patterns in conditions like endometriosis or premature ovarian insufficiency.

As tissue clearing technologies have advanced and become more aligned with cutting-edge imaging and machine learning tools, their widespread use is anticipated to greatly increase our comprehension of the complexities of the ovary and uterus. This integration will be vital for advancing female reproductive health and deepening our understanding of reproductive aging.
